# Examination of the Anti-Inflammatory, Antioxidant, and Xenobiotic-Inducing Potential of Broccoli Extract and Various Essential Oils during a Mild DSS-Induced Colitis in Rats

**DOI:** 10.1155/2013/710856

**Published:** 2013-02-28

**Authors:** Kristin Mueller, Nicole Michaela Blum, Andreas Stefan Mueller

**Affiliations:** Institute of Agricultural and Nutritional Sciences, Martin Luther University Halle-Wittenberg, Von-Danckelmann-Platz 2, 06120 Halle (Saale), Germany

## Abstract

Phytogenic compounds with antioxidant and anti-inflammatory properties are currently discussed as promising complementary agents in prevention and treatment of inflammatory bowel disease (IBD). Our study aimed to evaluate possible protective and curative effects of broccoli extract (BE) and of the essential oils of turmeric (Cuo), thyme (To), and rosemary (Ro) in a rat model with a mild dextran sulphate sodium- (DSS-) induced colitis. Therefore Wistar rats were fed a diet without an additive (Con) or diets with the addition of BE, Cuo, To, and Ro during the whole experiment. Pretreatment with Ro, Cuo, and To increased the expression of the tight junction protein Cldn3. All additives reduced mRNA of VCAM-1 which plays a crucial role in the first state of inflammatory response. Only Ro pretreatment affected the expression of the antioxidant enzymes HO1, GPx2, and of glutathione-S-transferases. All additives counteracted the DSS-induced rise in COX2 and VCAM-1 expression. Colonic IL-10 was increased by Cuo, To, and Ro. During the recovery phase DSS pretreatment increased NF**κ**B, VCAM-1, and MCP-1: This response was counter-regulated by all additives. We conclude that the phytogenic additives tested have a promising anti-inflammatory potential in vivo and a particular role in the prevention of IBD.

## 1. Introduction

Inflammatory bowel disease, including ulcerative colitis (UC) and Crohn's disease (CD), is a multifactorial relapsing-remitting disorder, characterized by intermittent periods of acute inflammation in the small and in the large intestine. The main difference between CD and UC is the location and the nature of the inflammatory changes. CD can affect any part of the gastrointestinal tract, from mouth to anus, although the onset of the majority of cases is located in the terminal ileum. In contrast UC is restricted to the colon and the rectum. The exact pathogenic mechanisms provoking both disorders remain almost unclear. However, in a number of cases overreactions of the immune system due to inflammatory stimuli can be observed. In this context, proinflammatory immune modulators like interleukin 1 beta (IL-1*β*), monocyte chemoattractant protein 1 (MCP-1), and vascular cell adhesion molecule 1 (VCAM-1) play an important role in the development of the disease [1, 2]. Nuclear factor “kappa-light-chain-enhancer” of activated B cells (NF*κ*B) represents a key transcription factor regulating the synthesis of genes involved in immune reactions and inflammatory response. In noninflamed tissues NF*κ*B is inhibited through linkage to its cytosolic inhibitor protein kappa B (I*κ*B). The activation of proteasomal I*κ*B degradation via phosphorylation of critical serine residues by proinflammatory stimuli elicits NF*κ*B translocation to the nucleus and the subsequent induction of its target genes like tumor necrosis factor (TNF)-*α* and other inflammatory mediators (interleukin 2, interleukin 6, interleukin 8, VCAM-1, intracellular cell adhesion molecule 1, and interferon *γ*) [3–5].

Dysfunction of the gut barrier accompanied by an increased intestinal permeability is another characteristic symptom in the pathophysiology of IBD [6]. As a consequence of the disordered permeability both antigenic determinants derived from food digestion and commensal or pathogenic bacteria can overcome the mucosal barrier unimpeded and provoke a continuous intestinal immune response and tissue damage [7]. In this context the increased permeability of gastrointestinal epithelial cells frequently results from the destruction of tight junctions. This process is triggered by oxidative stress deriving from reactive oxygen species (ROS), mycotoxins (e.g., patulin), bacterial components (e.g., lipopolysaccharides, LPS), and inflammatory mediators (e.g., cytokines) [8–11].

Due to their generally accepted antioxidant and anti-inflammatory properties, the use of plant extracts, and in particular of essential oils, represents a promising approach to prevent and cure IBD. For instance, the essential oil of *Origanum vulgare*, containing high concentrations of the phenolic terpenes carvacrol and thymol, has been shown to efficiently reduce the mRNA levels of the proinflammatory cytokines TNF*α*, IL-1*β*, and IL-6 in human THP-1 macrophages. In contrast in this study the anti-inflammatory cytokine IL-10 was significantly reduced [12]. A blend of oregano, anise, and lemon peel even could be demonstrated to evolve anti-inflammatory effects in piglets in vivo [13]. Data from current literature suggest that anti-inflammatory effects of plant extracts base on their direct and indirect antioxidant properties, which again depend on the chemical compounds contained in different plant extracts. The essential oils of thyme and oregano, which mainly contain the antioxidant terpene compounds thymol and carvacrol [14, 15], could be demonstrated to impair the mRNA and the protein concentration of the pro-inflammatory cytokines IL-1*β* and IL-6 in mice with 2,4,6-trinitrobenzol (TNBS-) induced colitis [16]. Carnosol, a terpene of rosemary oil in vitro, showed indirect antioxidant effects via inducing nuclear factor erythroid 2-related factor 2- (Nrf2-) regulated antioxidant enzyme expression and additionally decreased pro-inflammatory mediators like NF*κ*B, TNF*α*, IL-1*β*, IL-6, COX2, and ICAM-1 [17–19]. In another in vitro study, treatment with sulforaphane, an isothiocyanate mainly contained in broccoli, decreased the mRNA concentration of the pro-inflammatory cytokines TNF*α* and IL-1*β* in murine RAW264.7 macrophages due to a pro-inflammatory stimulus with bacterial LPS. In this experiment the reduction of inflammation was accompanied by an increase in the expression of the antioxidant enzyme HO1 via the Nrf2/Kelch-like ECH-associated protein 1 (Keap1) pathway [20]. Similar strong indirect antioxidant effects through the modification of Keap1 sulfhydryl groups have also been described for ar-turmeron, the main terpene of curcuma oil. In addition ar-turmeron possesses also direct antioxidant properties [21].

However, until today studies comparing the anti-inflammatory potential of different phytogenic substances due to a pro-inflammatory stimulus are not available. DSS is a chemical compound routinely used to induce a colitis in model animals and therefore to mimic similar inflammatory conditions as present in IBD.

Consequently our study aimed to investigate the connection between the antioxidant potential and anti-inflammatory effects of broccoli extract, turmeric oil, thyme oil, and rosemary oil in rats with a mild DSS-induced colitis.

## 2. Materials and Methods

### 2.1. Animals and Diets

The protocol of the rat study was approved by the Regional Council of Halle and by the Animal Welfare Committee of the Martin Luther University Halle-Wittenberg (record token: 42502-2-1093-MLUG). 92 four=week old male Wistar rats (mean body weight: 186.2 ± 9.45 g) were obtained from Harlan laboratories (Horst, The Netherlands). The rats were fed a standard diet without phytogenic feed additives for an acclimatisation period of 14 days.

At an age of 6 weeks and a mean live weight of 231.0 ± 12.8 g the rats were assigned to 6 experimental groups of 16 rats each. During the following experimental periods the control groups (Con and DSS) were fed a basal diet that met the nutritional demands of the NRC for growing rats. This basal diet contained no phytogenic additive (Table 1). 

The diet of group BE contained 8750 mg/kg broccoli sprouts extract (JARROW Formulas). 1494 mg/kg diet of *Curcuma longa *oil (Cuo), 618 mg/kg of *Thymus vulgaris *oil (To), and 680 mg/kg of *Rosmarinus officinalis* oil (Ro) were added to the diets of the other groups in order to standardize the concentration of the isothiocyanate sulforaphane (BE) and of the individual main terpenes (Cuo, To, and Ro) to a value of 2 mmol/kg diet. The main terpenes considered were ar-turmerone for Cuo, thymol for To, and 1,8-cineol for Ro. All diets were pelleted with a pellet mill using an 8 mm die and fed during the whole course of the experiment. The rats had free access to their respective diet and to tap water. Lighting, humidity, and temperature regime was in accordance with the recommendations of the Society for Laboratory Animal Science (2004) [22]. The trial consisted of 3 phases: (1) pretreatment phase (phase 1: 7 days), (2) DSS-treatment phase (phase 2: 6 days), and (3) recovery phase (phase 3: 6 days).  Table 2 overviews the feeding protocol in detail.

The animal model of a DSS-induced colitis was chosen due to several histological and biochemical similarities with human IBD [23, 24]. In the DSS-treatment phase, 4% DSS (40 kDa; Sigma-Aldrich) was administered via drinking water to all rats for 6 days, with the exception of the Con group, in order to induce a mild intestinal inflammation. Feed intake and individual live weight were recorded after one week, daily during the DSS-phase and every other day during the recovery phase.

At the end of phase 1 four rats were killed for organ sampling (liver and colon), and after phases 2 and 3 six rats per group were sacrificed. For the histological examination and for the determination of relative mRNA concentrations of antioxidant and xenobiotic enzymes and of inflammation parameters, colon samples were prepared from a 10 cm segment distal to the caecum. Liver samples were excised from the middle of lobus sinister lateralis.

During treatment with 4% DSS the disease activity index (DAI) was used to assay the severity of the induced colitis. DAI was determined daily in phase 2 and every other day in phase 3 (recovery phase). The scoring system was based on body weight loss, stool consistency, and macroscopic fecal blood debris. For each mentioned parameter a scale ranging from 0 to 4 was applied as described previously [25].

### 2.2. Colonic Histology

For the histological examination freshly dissected colon samples were washed with 0.9 (w/v) % NaCl and cryoconserved in a freezing medium (Jung; Leica Instruments, Nussloch, Germany). Serial cross-sections (7 *μ*m) were prepared using a microtome (CM 1850 UV microtome, Jung; Leica) and fixed on sterile usual microscope slides. After staining the samples with Haematoxylin-Eosin full-thickness slices tissue architecture, infiltration of neutrophilic granulocytes into the mucosa and into the submucosa, and the formation of crypt abscesses were examined under an inversion microscope using a blind protocol.

### 2.3. RNA Preparation and Real-Time RT-PCR Analysis

Total RNA from 100 mg of liver and colon tissue was isolated using the acid guanidinium thiocyanate-phenol-chloroform extraction method [26]. Most recently a strong DSS RNA interference during acute DSS-treatment resulting in the lack of signals during gene expression analysis has been described [27]. To assure the correctness of gene expression analyses in our experiment polyA+ mRNA from the colonic samples of the DSS-phase was purified using the GenElute mRNA Miniprep Kit (Sigma-Aldrich, MO, USA) according to the manufacturers' protocol. Following the photometrical determination of RNA concentration and purity at 260 nm and 280 nm, reverse transcription of 3.0 *μ*g of total RNA or of 0.3 *μ*g of purified mRNA and real-time RT-PCR were performed as described previously [28]. Gene bank accession numbers and primer sequences (5′ → 3′) are shown in Table 3. 

Gene specific mRNA expression was analyzed with the Rotor-Gene 6000 series software using the ΔΔCt method [29]. The amplification data of the single genes were normalized to the expression of the two most stable reference genes in each tissue (liver: *β*-actin, ribosomal protein L13A (Rpl13a); colon: Rpl13a, hypoxanthine phosphoribosyltransferase 1 (Hprt1)). Relative mRNA expression levels are expressed as x-fold changes relative to group Con = 1.0.

### 2.4. Statistical Analysis

Data are presented as means ± their standard error of the mean (SEM). Statistical differences were analyzed with SPSS 19.0 for Windows (IBM, Chicago, USA) using one-way ANOVA after verifying the normality of distribution (Shapiro Wilk test and Kolmogorov Smirnov test) and the homogeneity of variances (Levene test). The Least Significant Difference test (LSD) was used to analyze significant differences between means if variances were homogenous. If not, the Games Howell test was used. At an error probability of less than 5% (*P* < 0.05) differences between means were considered as statistically significant.

## 3. Results

### 3.1. Body Weight Development

Neither food intake (data not shown) nor final body weight (Table 4) of the rats was influenced significantly in the different experimental phases by feeding the specific diets tested. Remarkably, also DSS treatment in the second phase did not affect the above-mentioned parameters significantly. 

### 3.2. Colitis Severity by Disease Activity Index (DAI)

The course of the mild colitis, induced by the administration of 4% DSS to rats for 6 days, was controlled daily in phase 2 by measuring the DAI. A high number of rats from all DSS-treated groups had a soft stool consistency, but severe diarrhea accompanied by blood debris could not be observed. Moreover a distinct weight loss, frequently observed under DSS treatment, was present only in some cases and only for one day. Consequently the DAI in all DSS-treated groups remained below 1.0 (DSS: 0.12, BE: 0.10, Cuo: 0.10, To: 0.07, and Ro: 0.13) and did not differ significantly from Con rats receiving no DSS. In phase 3 obvious changes in stool consistency almost disappeared or were much less pronounced than in phase 2.

### 3.3. Histology

**Figure 1:**
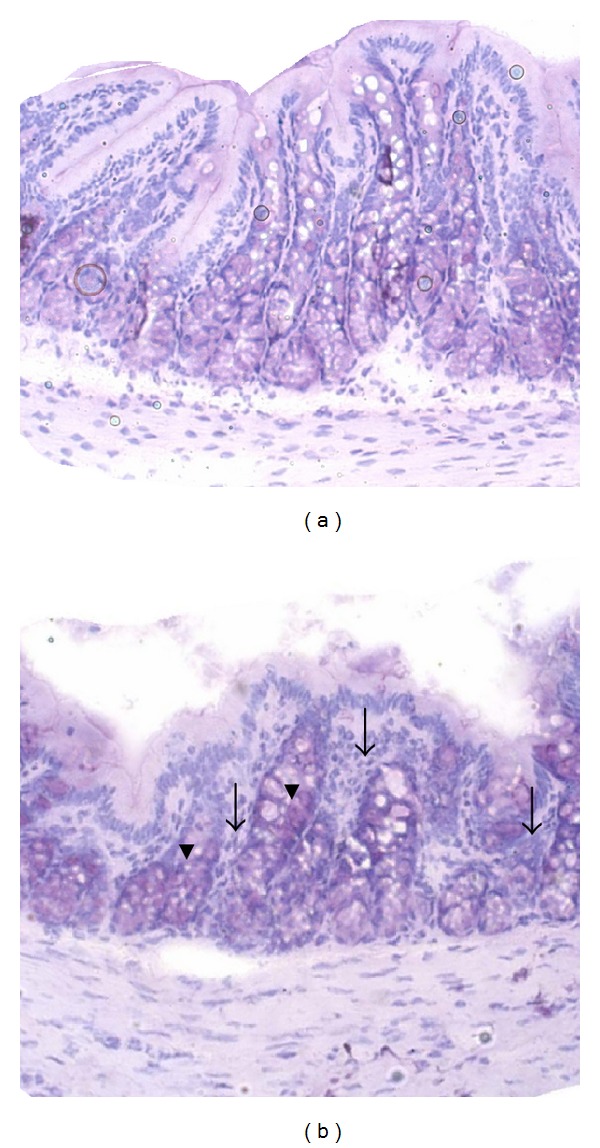
Representative pictures of proximal colon sections from healthy Con rats (a) and DSS-treated rats (b) fed the control diet. Haematoxylin and Eosin (H and E) stained colon cross-sections from phase 2 were analyzed with inversion microscopy (20x). Arrows indicate increased leucocyte infiltration, and arrowheads indicate the loss of goblet cells caused by DSS treatment (b).

**Figure 2:**
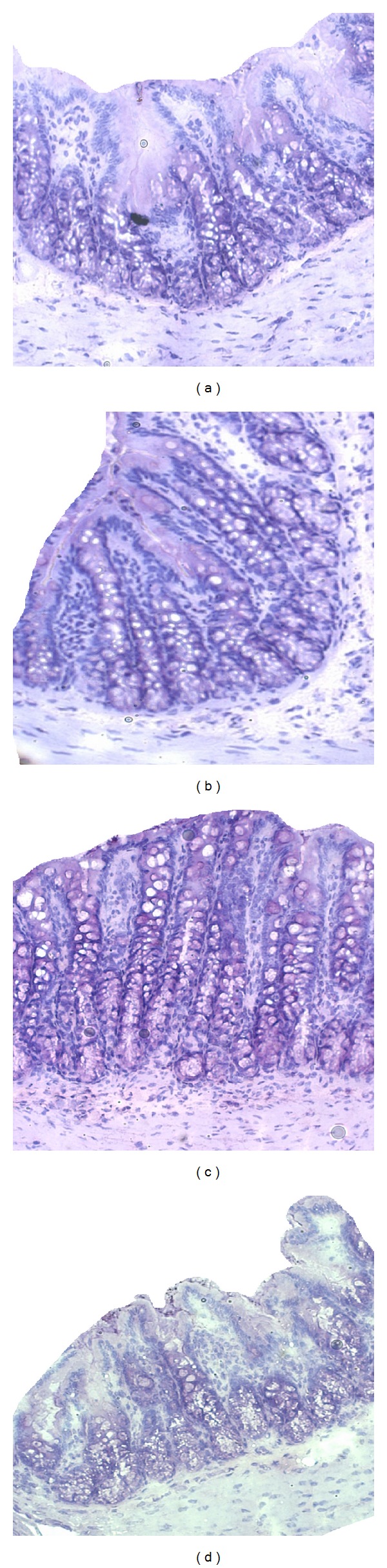
Representative pictures of proximal colon sections from DSS-treated rats fed experimental diets with broccoli extract (a), turmeric oil (b), thyme oil (c), or rosemary oil (d). Haematoxylin and Eosin (H and E) stained colon cross-sections from phase 2 were analyzed with inversion microscopy (20x).

DSS treatment caused no significant observable macroscopic changes in colonic tissue architecture between the experimental groups, including the untreated Con rats. Furthermore microscopic analysis revealed no significant histological damage to the colonic mucosa of rats exposed to 4% DSS for 6 days. However, DSS treatment tended to accelerate initial damage to the mucosa, characterised by the loss of goblet cells and the occurrence of a more diffuse crypt architecture compared to the colon of healthy Con rats. Additionally, the accumulation of neutrophils, infiltrating the lamina propria could be observed more frequently in colonic slices of DSS-treated rats. These mentioned DSS-induced mucosal alterations are shown in Figure 1. DSS treatment in combination with the tested phytogenic extracts also caused slight mucosal damage (Figure 2), but the severity seemed to be much lower than in group DSS (Figure 1(b)).

### 3.4. mRNA Expression of NF*κ*B, TNF*α*, and Various Inflammatory-Mediating Enzymes in Colon and Liver Tissue

The analyzed mRNA expression patterns of pro- and anti-inflammatory genes controlled by NF*κ*B and TNF*α* differed among the experimental periods and the tissues investigated (Tables 5 and 6). During the 7-day pretreatment phase 1 the mRNA abundance of the colonic pro-inflammatory markers COX2 and IL-1*β*, of the anti-inflammatory cytokine IL-10, of the cell adhesion molecules MCP-1 and VCAM-1, and of the tight junction protein Cldn3 showed a high intraindividual variance. In contrast in phase 1 of the trial liver mRNA data of the above-mentioned genes showed a much better homogeneity. Nevertheless, feeding diets with BE-, Cuo-, To-, and Ro-addition reduced colonic VCAM-1 mRNA by 57 to 64% compared to untreated control rats. This reduction was significant for Cuo, To, and Ro and represented a trend (*P* < 0.10) for BE. Moreover in phase 1 colonic Cldn3 expression was significantly higher in Ro-treated rats and tendencially higher in Cuo- and To-treated rats than in their Con littermates.

In phase 1 feeding diets containing BE, Cuo, and Ro significantly decreased liver mRNA abundance of the pro-inflammatory cytokine IL-10 by 32 to 44%.

Following treatment with an intermediate dose of 4% DSS for 6 days (phase 2) specific effects of the phytogenic substances on inflammatory markers could be analyzed in the colon, whereas these parameters remained uninfluenced in the liver. DSS treatment had no significant influence on colonic NF*κ*B mRNA expression. In contrast TNF*α* expression increased in all DSS-treated groups, including those receiving phytogenic additives, compared to untreated control rats. This pro-inflammatory response was also reflected by an 18-fold increase in colonic COX2 mRNA expression in DSS-treated rats receiving no phytogenic additive compared to untreated Con rats. Feeding diets containing BE, Cuo, and Ro reduced COX2 expression nearly to the level in untreated controls. To addition lowered COX2 expression somewhat less than the other additives. Nevertheless also DSS-treated To rats had a colonic COX2 expression, which was tendencially lower than in their DSS-treated littermates receiving no phytogenic additive. Whereas the colonic mRNA concentration of MCP-1 was not lowered by phytogenic feed additives during the DSS period, all additives tested, reduced VCAM-1 expression by 73 to 83%, and compared to DSS-treated control rats. DSS treatment generally increased the expression of the anti-inflammatory IL-10 in comparison to Con rats without DSS treatment. This effect was relatively small in DSS-treated rats receiving no phytogenic additive and in those receiving the diet containing BE. In contrast the impact of Cuo, To, and Ro addition on colonic IL-10 expression was significant. In all DSS-treated rats colonic Cldn3 mRNA decreased compared to untreated control rats. This reduction of Cldn3 expression tended to be lower in DSS-treated rats without an additive and in the DSS-treated Cuo group, and it was significant in their DSS-treated littermates receiving diets with the addition of BE, To, and Ro. 

In the final recovery period (phase 3) NF*κ*B mRNA strongly increased in the colon of DSS-treated control rats compared to their untreated littermates. All phytogenic additives lowered NF*κ*B response considerably (*P* < 0.10). These characteristic changes in NF*κ*B mRNA were reflected by similar changes in the expression of the pro-inflammatory cytokine IL-1*β*. Nevertheless, in all DSS-treated rats, including those receiving phytogenic additives, colonic TNF*α* mRNA levels remained significantly higher throughout the recovery period. In the recovery period also liver TNF*α* mRNA level was increased in DSS-treated rats without a phytogenic additive, but not in rats fed diets containing any phytogenic substance, compared to untreated controls. In the recovery period colonic COX2 mRNA levels of DSS-treated control rats dropped nearly to the level in untreated controls, and they were further decreased in DSS rats receiving phytogenic additives compared to phase 2 of the experiment. In phase 3 colonic mRNA levels of MCP-1 and VCAM-1 in DSS-treated rats without a phytogenic additive further increased compared to phase 2, and they were significantly higher than in untreated controls. Interestingly a similar effect could be observed in rats fed a BE containing diet. In contrast rats fed diets containing the other additives (Cuo, To, and Ro) showed MCP-1 and VCAM-1 mRNA levels, which were comparably low as in untreated controls and significantly reduced compared to DSS controls. However, in the liver neither DSS treatment nor combining DSS treatment with feeding phytogenic additives had an influence on MCP-1 and VCAM-1 mRNA abundance in the recovery phase. In contrast to the acute DSS-treatment period, Cldn3 levels were not lower in the DSS-treated groups than in untreated controls. In rats receiving the To diet Cldn3 mRNA was even 1.5–2.0-fold higher than in the other groups.

### 3.5. mRNA Expression of Nrf2, Keap1, and Various ARE-Regulated Enzymes in the Colon

The Nrf2/Keap1 system regulates the expression of antioxidant enzymes and xenobiotic enzymes. In the current experiment some characteristic changes could be observed with regard to Nrf2-, and Keap1 expression and on the expression of ARE-regulated antioxidant and phase II enzymes, depending on the experimental phase and on the treatment of the rats (Table 7). In the pretreatment phase the high impact of Ro on colonic Keap1 expression was directly reflected by an increase in the expression of several ARE-regulated antioxidant enzymes like HO1 and GPx2 and on phase II enzymes like GSTK1, P1, and T2.

In the initial phase all other additives tested had neither a distinct and directed influence on Keap1 mRNA nor on the expression of the above-mentioned target genes. During acute DSS treatment the effects on ARE-regulated enzymes were relatively small. Whereas the antioxidant enzymes HO1 and NQO1 tended to be higher in DSS-treated control rats than in untreated controls, all phytogenic additives reduced this particular DSS effect to a little extent. In contrast the expression of the ARE-regulated phase II enzymes GSTK1, P1, and T2 tended to be lowered by DSS treatment. In these cases Nrf2 mRNA abundance showed a similar alteration like the above-mentioned phase II enzymes. The reduction of GSTK1, P1, and T2 was slightly aggravated by the phytogenic additives. In the recovery phase, in all DSS-treated groups, GPx2 expression decreased below the expression in untreated control rats. A differential development could be analyzed for the different GST subclasses. Whereas the mRNA expression of GSTK1 and P1 in DSS-treated rats remained below the level of their untreated littermates, the mRNA concentration of GST T2 in treated rats exceeded that of untreated. In the liver no gross changes could be observed with regard to the expression of the antioxidant and phase II enzymes investigated due to the different dietary and pro-inflammatory conditions.

## 4. Discussion

Our current trial aimed to investigate the impact of different phytogenic substances on changes in the expression of genes related to inflammation and of ARE-regulated antioxidant and phase II enzyme genes in rats previous to (phase 1), during (phase 2) and subsequent (phase 3) to the provocation of an experimental colitis with DSS. 

The use of 4% DSS was intended to induce a mild colitis and therefore to reflect the onset of an acute local gut inflammation. Interestingly, DSS application caused no significant loss of body weight, neither in the DSS-treatment phase nor in the recovery phase. These data are consistent with observations from the current literature [30, 31]. Moreover Hakansson et al. [31] also showed only small alterations in female rats' DAI due to acute treatment with 4% DSS for 7 days. Accordingly, in the mentioned study colitis symptoms completely disappeared in the recovery phase.

In contrast other studies using an equal or even a lower DSS concentration reported a high weight loss, a strongly increased DAI, and severe damage to the colonic mucosa [32–35]. The colitis inducing potential of DSS may depend on a number of factors including (1) the DSS concentration and the exact molecular weight of the DSS compound used, (2) the duration of DSS-exposition, and (3) the species and the gender of the experimental animals.

Our results for DAI confirmed the findings of the histological examination, in which only small alterations in mucosal architecture could be found. In summary these findings indicated a mild colitis as intended in our experiment. In contrast other investigations in the current literature reported on an extensive mucosal damage accompanied by crypt loss, ulcerations and erosion by treating rats with 4% DSS [34–36]. Nevertheless, also in our trial DSS-treated rats had a higher infiltration of neutrophils in the lamina propria and small alterations in crypt architecture. However, a significant crypt loss and mucosal damage could not be observed. These differences may result from species differences due to DSS treatment, from differences in the DSS concentration used, and from differences in the DSS-application period [37, 38]. 

To the best of our knowledge currently there exist no comparable studies investigating the development of the above mentioned parameters under nonstimulated conditions as represented by phase 1 of our trial. 

In the pretreatment phase of our experiment Ro turned out to be the most efficient phytogenic substance with regard to the upregulation of the ARE-regulated phase II enzymes GSTK1, P1, and T2 and of the ARE-regulated antioxidant enzyme GPx2. These changes were directly accompanied by a significant increase in Keap1 mRNA. 

The strong effects of Ro on phase II enzymes containing an ARE promoter confirm the results of studies from our group carried out with growing chickens and growing pigs [39, 40]. In contrast, in these studies also BE, Cuo, and To effected a significant increase in the intestinal mRNA levels of the above mentioned gene family. The deviation from these results may presumably derive from the shorter prefeeding period in our current study (1 week versus 5 weeks in the chicken trial and 4 weeks in the piglet trial). Moreover also the results of a very recent rat trial of our group corroborate this “time hypothesis.” In the mentioned study feeding of BE in combination with different dietary selenium concentrations to rats increased the mRNA of a broad spectrum of colonic ARE-regulated antioxidant and phase II enzymes about 3.5-fold. In the latter mentioned trial we have moreover suggested that on the basis of its mRNA expression, the cytosolic Nrf2 adapter protein Keap1 seems to be a more sensitive indicator of ARE driven gene expression than the transcription factor Nrf2 per se [41]. 

Even though effects on inflammation markers were not significant in all cases in our current trial, all phytogenic substances tested had an overall positive influence on inflammation related parameters. For instance, all additives reduced colonic COX2 expression, and they increased the anti-inflammatory cytokine IL-10 considerably.

Moreover VCAM-1 expression was decreased by all plant extracts tested to a high extent. This particular result is in accordance with the data of a current in vitro study [42]. Moreover in our trial the mRNA expression data for colonic Cldn3 have shown for the first time that the essential oils of turmeric, thyme, and rosemary may improve the mucosal barrier function by the upregulation of this tight junction protein. Comparable beneficial effects of plant extracts on tight junction proteins have been described only for two other plant extracts, namely, berberine and apple extracts until today [43, 44].

Treatment with 4% DSS for 6 days in our trial has caused a mild colonic inflammation as indicated by the distinct increase in the expression of pro-inflammatory parameters like COX2 and TNF*α* in group DSS compared to healthy Con rats. Although a rise in the expression of these pro-inflammatory mediators could also be observed in the DSS groups, receiving plant extracts, it was considerably less pronounced, suggesting anti-inflammatory effects of broccoli extract, turmeric oil, thyme oil, and rosemary oil during the acute phase of a mild colitis. With regard to this aspect our data confirm the results of current in vitro and in vivo studies describing beneficial effects of plant extracts during acute phlogistic processes via the reduction of pro-inflammatory mediators [12, 16, 20, 45–49]. However, reduced Cldn3 mRNA level in all DSS-treated rats may indicate that none of the plant extracts investigated could prevent changes in tight junctions during acute DSS exposition.

In contrast to the data of Reed et al. [50], showing the activation of NF*κ*B subsequent to a 6-day treatment period with 5% DSS, in our trial a DSS-dependent increase in the mRNA expression of NF*κ*B and of its targets TNF*α*, IL-1*β*, MCP-1, and VCAM-1 did not occur until the end of phase 3 (6 days after final DSS treatment). These results may derive from the lower DSS concentration (4%) used in our trial. With the exception of group To in phase 3 no changes in Cldn3 mRNA expression could be observed in the other experimental groups compared to Con rats, suggesting that in particular To may improve intestinal barrier function [51].

**Figure 3:**
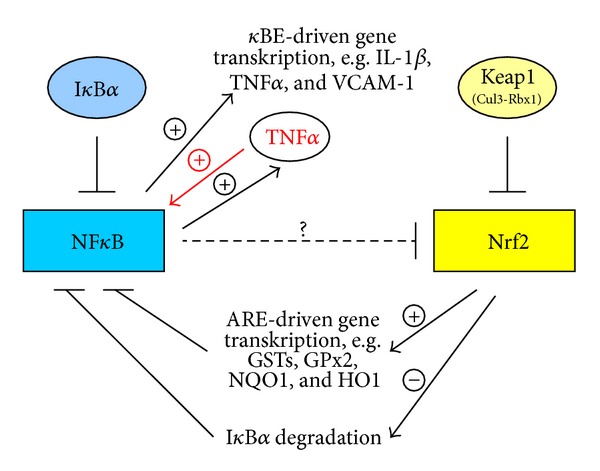
NF*κ*B and Nrf2 crosstalk under balanced anti- and prooxidant conditions.

**Figure 4:**
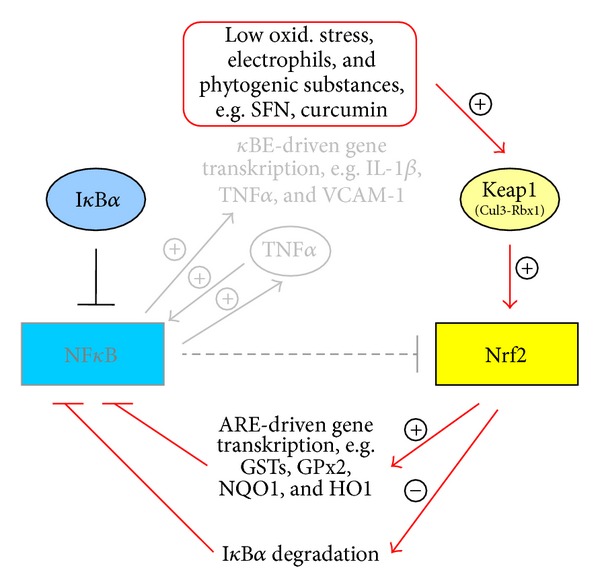
NF*κ*B and Nrf2 interaction due to feeding phytogenic compounds or low oxidative stress.

**Figure 5:**
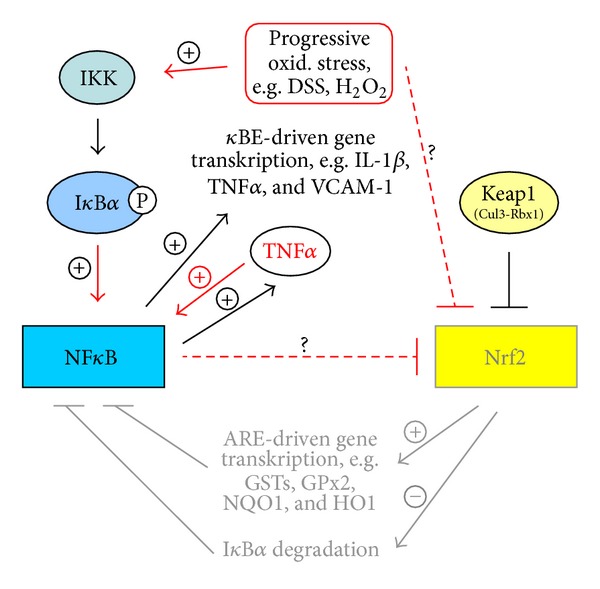
NF*κ*B and Nrf2 interaction due to DSS treatment or progressive oxidative stress.

Data from current literature suggest that the transcription factor Nrf2, responsible for the induction of antioxidant and xenobiotic enzymes, is of importance in the control of NF*κ*B dependent inflammatory processes (Figure 3) [52]. Vice versa it is speculated that an increase in NF*κ*B may inhibit Nrf2 signaling. Several well-known Nrf2 activators, for example, curcumin, resveratrol, and sulforaphane, are believed to suppress LPS- and DSS-induced NF*κ*B activation [53–56], whereas the direct relationship between Nrf2 manipulation and NF*κ*B-inhibition has not been proven until today. Our results for the acute inflammatory phase 2 cannot confirm an association between Nrf2 and NF*κ*B. However, a distinct negative association exists between Nrf2 and TNF*α*, which again is believed to be the strongest first trigger of NF*κ*B activation (Figures 3–5) [57]. Thus due to acute DSS exposition Nrf2 expression was distinctly decreased, whereas TNF*α* expression was strongly elevated. In contrast no differences in the relation between the mentioned transcription factors existed under noninflamed conditions (phase 1). Moderate oxidative stress seems to activate Nrf2 and its target genes accompanied by the simultaneous inhibition of NF*κ*B signaling (Figure 4). This hypothesis seems to be confirmed by the general decrease of VCAM-1 mRNA (NF*κ*B target) through application of all plant extracts and the increase in GSTK1 and T2 mRNA (Nrf2 targets) by Cuo and Ro application in phase 1 of our study (Figure 4).

In phase 2 DSS treatment alone obviously has induced considerable oxidative stress in the colon accompanied by a significant inhibition of Nrf2 expression, finally leading to an increase in NF*κ*B mRNA in phases 2 and 3 of our trial (Figure 5). In contrast phytogenic additives seem to attenuate both NF*κ*B induction and the upregulation of its pro-inflammatory targets. This phenomenon may be the result of the rise in Nrf2-dependent antioxidant and xenobiotic enzymes. 

Our liver data indicate that DSS-dependent inflammatory processes seem to be restricted to the colon initially, but that they can also provoke a systemic inflammatory response as indicated by the increase in TNF*α* expression in DSS-treated rats in phase 3.

## 5. Conclusions

Our experimental design has allowed for the simultaneous examination of (1) preventive effects of various plant extracts on intestinal health (phase 1), of (2) their anti-inflammatory and antioxidant potential during acute DSS-induced colitis (phase 2), and of (3) their influence on parameters related to inflammation and the antioxidant system during the recovery process (phase 3). 

We conclude the following.

- The treatment of rats under noninflamed conditions with broccoli extract and the essential oils of turmeric, thyme, and rosemary promotes intestinal health by reducing the pro-inflammatory adhesion molecule VCAM-1 and by increasing the tight junction marker Cldn3 leading to an improved gut barrier.
- The phytogenic additives investigated have anti-inflammatory properties as indicated by the reduction of the DSS-induced increase in pro-inflammatory mediators like NF*κ*B, VCAM-1, MCP-1, and COX2 to a greater or lesser extent.

Further research is needed to evaluate the role of phytogenic additives in modulating NF*κ*B-Nrf2 interactions in more detail. Moreover detailed investigations with regard to the time-dependent regulation of inflammatory and antioxidant responses during an acute gut inflammation are urgently needed. Nevertheless, plant extracts and in particular essential oils may represent promising substances in the complementary therapy of IBD with a particular focus in the prevention.

## Figures and Tables

**Table 1:** Basal diet.

Ingredient	g/kg basal diet
Wheat (DEUKA GmbH und Co. KG, Könnern, Germany)	237.9
Maize (DEUKA GmbH und Co. KG, Könnern, Germany)	200.0
Barley (DEUKA GmbH und Co. KG, Könnern, Germany)	156.0
Soybean meal, 46% CP (DEUKA GmbH und Co. KG, Könnern, Germany)	220.0
Wheat bran (DEUKA GmbH und Co. KG, Könnern, Germany)	78.8
Oat (DEUKA GmbH und Co. KG, Könnern, Germany)	69.0
Sun flower oil	15.0
Lysine (Feed Grade, China)	0.3
dl-methionine (Degussa, Duesseldorf, Germany)	2.0
Vitamin and mineral premix	12.1
Calcium carbonate (Sigma-Aldrich)	2.5
Calcium phosphate (Mischfutter und Landhandel GmbH, Edderitz, Germany)	7.9

**Table 2:** Feeding protocol.

Group	Phytogenic additive	Koncentration per kg diet	Phase 1	Phase 2	Phase 3
Con	None	—	7 days diets and water ad libitum	6 days diets and water ad libitum	6 days diets and water ad libitum
DSS	None	—	7 days diets and water ad libitum	6 days diets ad libitum and 4% DSS	6 days diets and water ad libitum
BE	Broccoli extract	2 mmol sulforaphane	7 days diets and water ad libitum	6 days diets ad libitum and 4% DSS	6 days diets and water ad libitum
Cuo	Turmeric oil	2 mmol ar-turmerone	7 days diets and water ad libitum	6 days diets ad libitum and 4% DSS	6 days diets and water ad libitum
To	Thyme oil	2 mmol thymol	7 days diets and water ad libitum	6 days diets ad libitum and 4% DSS	6 days diets and water ad libitum
Ro	Rosemary oil	2 mmol 1,8-cineol	7 days diets and water ad libitum	6 days diets ad libitum and 4% DSS	6 days diets and water ad libitum

**Table 3:** Gene bank accession numbers and primer sequences of the genes investigated by real-time RT-PCR.

Gene name (abbreviation used)	Gene bank accession number	Primer sequences (5′ → 3′) for = forward; rev = reverse;
Chemokine (C-C motif) ligand 2 (Ccl2) (MCP1)	NM_031530	for: GTGCGACCCCAATAAGGAA
		rev: TGAGGTGGTTGTGGAAAAGA
Claudin 3 (Cldn3)	NM_031700	for: TATCCTACTGGCAGCCTTCG
		rev: GTTCCCATCTCTCGCTTCTG
Copper/zinc superoxide dismutase (SOD1)	NM_017050	for: CCACTGCAGGACCTCATTTT
		rev: CACCTTTGCCCAAGTCATCT
C-reactive protein (CRP)	NM_017096	for: GTCTCTATGCCCACGCTGAT
		rev: CCGTCAAGCCAAAGCTCTAC
Glutathione S-transferase K1 (GSTK1)	NM_181371	for: GAGCATGGAGCAACCAGAGAT
		rev: AGCTTGCTCTTCACCAGTTCG
Glutathione S-transferase P1 (GSTP1)	NM_012577	for: GAGGCAAAGCTTTCATTGTGG
		rev: GTTGATGGGACGGTTCAAATG
Glutathione S-transferase T2 (GSTT2)	NM_012796	for: GAGGAAAAGGTGGAACGGAAC
		rev: CGCCCCTCAAACAGATTACAG
Glutathione peroxidase 2 (GPx2)	NM_183402	for: GTGTGATGTCAATGGGCAGAA
		rev: ACGTTTGATGTCAGGCTCGAT
Heme oxygenase 1 (HO1)	NM_012580	for: AGGCACTGCTGACAGAGGAAC
		rev: AGCGGTGTCTGGGATGAACTA
Hypoxanthine phosphoribosyltransferase 1 (Hprt1)	NM_012583	for: GCAGACTTTGCTTTCCTTGG
		rev: TCCACTTTCGCTGATGACAC
Interleukin 1 beta (IL-1*β*)	NM_031512	for: CTGTGACTCGTGGGATGATG
		rev: GGGATTTTGTCGTTGCTTGT
Interleukin 10 (IL-10)	NM_012854	for: CTGGAGTGAAGACCAGCAAAGG
		rev: GGAGAAATCGATGACAGCGTCG
Kelch-like ECH-associated protein1 (Keap1)	NM_057152	for: GTGGCGGATGATTACACCAAT
		rev: GAAAAGTGTGGCCATCGTAGC
NAD(P)H dehydrogenase [quinone] 1 (NQO1)	NM_017000	for: CGCAGAGAGGACATCATTCA
		rev: CGCCAGAGATGACTCAACAG
Nuclear factor (erythroid-derived 2)-like 2 (Nrf2)	NM_031789	for: CCAAGGAGCAATTCAACGAAG
		rev: CTCTTGGGAACAAGGAACACG
Nuclear factor kappa B (NF*κ*B)	L26267	for: CTTCTCGGAGTCCCTCACTG
		rev: CCAATAGCAGCTGGAAAAGC
Prostaglandin-endoperoxide synthase 2 (Ptgs2) (COX2)	NM_017232	for: GCTGTACAAGCAGTGGCAAA
		rev: CCCCAAAGACAGCATCTGGA
Ribosomal protein L13A (Rpl13a)	NM_173340	for: CCCTCCACCCTATGACAAGA
		rev: CCTTTTCCTTCCGTTTCTCC
Tumor necrosis factor alpha (TNF*α*)	NM_012675	for: GCCAATGGCATGGATCTCAAAG
		rev: AAATCGGCTGACGGTGTGGG
Vascular cell adhesion molecule 1 (VCAM 1)	NM_012889	for: TGACATCTCCCCTGGATCTC
		rev: CTCCAGTTTCCTTCGCTGAC
*β*-actin	NM_031144	for: ATCGTGCGTGACATTAAAGAGAAG
		rev: GGACAGTGAGGCCAGGATAGAG

**Table 4:** Body weight changes in DSS-treated rats fed with broccoli extract and various essential oils compared to an untreated control.

Period	Body weight in g											
	Con		DSS		BE		Cuo		To		Ro	
	MW	SEM	MW	SEM	MW	SEM	MW	SEM	MW	SEM	MW	SEM
start	229.4	4.05	232.3	2.45	230.9	3.20	231.2	3.10	230.7	3.58	231.8	2.85
day 7	266.0	7.80	263.4	6.48	280.0	3.75	273.5	6.90	265.0	2.13	281.0	0.70
day 13	297.7	5.72	300.0	5.92	297.3	3.26	297.5	4.21	295.4	4.19	301.1	4.13
day 19	325.2	6.74	321.1	8.16	323.4	4.04	322.1	5.92	321.1	3.96	320.5	9.14

Values are means ± SEM. *n* = 16 rats per group for phase 1 (start day 7), *n* = 12 rats per group for phase 2 (day 13), and *n* = 6 rats per group for phase 3 (day 19).

**Table 5:** Effects of feeding broccoli extract and various essential oils on relative mRNA expression of various pro- and anti-inflammatory parameters in the colon of DSS-treated rats compared to an untreated control.

Gene	Period	Experimental group compared				
		with untreated control rats				
		DSS	BE	Cuo	To	Ro
	Phase 1	—	*↔*	*↔*	*↔*	*↔*
NF*κ*B	Phase 2	*↔*	*↔*	*↔*	*↔*	*↔*
	Phase 3	↑	*↔*	*↔*	*↔*	*↔*
	Phase 1	—	*↔*	*↔*	*↔*	*↔*
TNF*α*	Phase 2	*↔*	*↔*	*↔*	↑	*↔*
	Phase 3	↑	↑	↑	↑	↑
	Phase 1	—	*↔*	*↔*	*↔*	*↔*
COX2	Phase 2	↑↑↑	*↔*	*↔*	↑	*↔*
	Phase 3	*↔*	*↔*	*↔*	*↔*	*↔*
	Phase 1	—	*↔*	*↔*	*↔*	*↔*
IL-1*β*	Phase 2	*↔*	*↔*	*↔*	*↔*	*↔*
	Phase 3	↑↑	*↔*	*↔*	*↔*	*↔*
	Phase 1	—	*↔*	*↔*	*↔*	*↔*
IL-10	Phase 2	*↔*	*↔*	↑	↑↑↑	↑↑
	Phase 3	n.d.	n.d.	n.d.	n.d.	n.d.
	Phase 1	—	*↔*	*↔*	*↔*	*↔*
MCP-1	Phase 2	*↔*	↑	↑	↑	↑
	Phase 3	↑	↑	*↔*	*↔*	*↔*
	Phase 1	—	*↔*	↓↓	↓↓	↓↓
VCAM-1	Phase 2	(↑)	(↓)	(↓)	(↓)	(↓)
	Phase 3	↑↑	↑	*↔*	*↔*	*↔*
	Phase 1	—	*↔*	*↔*	*↔*	↑
Cldn3	Phase 2	*↔*	↓↓	*↔*	↓↓	↓↓
	Phase 3	*↔*	*↔*	*↔*	↑	*↔*

Different arrows summarize the effects on gene expression: *↔* no effect, ↑: increase, ↓: decrease, ( ): significant results compared to group DSS, and n.d.: not detectable. Means ± SEM and the results of statistical analysis are presented in the appendix.

**Table 6:** Effects of feeding broccoli extract and various essential oils on relative mRNA expression of various pro- and anti-inflammatory parameters in the liver of DSS-treated rats compared to an untreated control.

Liver	Period	Experimental group compared				
		with untreated control rats				
Gene		DSS	BE	Cuo	To	Ro
	Phase 1	—	*↔*	*↔*	*↔*	*↔*
NF*κ*B	Phase 2	*↔*	*↔*	*↔*	*↔*	*↔*
	Phase 3	*↔*	*↔*	*↔*	*↔*	*↔*
	Phase 1	—	*↔*	*↔*	*↔*	*↔*
TNF*α*	Phase 2	*↔*	*↔*	*↔*	*↔*	*↔*
	Phase 3	↑	*↔*	*↔*	*↔*	*↔*
	Phase 1	—	*↔*	*↔*	*↔*	*↔*
COX2	Phase 2	*↔*	*↔*	*↔*	*↔*	*↔*
	Phase 3	n.d.	n.d.	n.d.	n.d.	n.d.
	Phase 1	—	*↔*	*↔*	*↔*	*↔*
IL-1*β*	Phase 2	*↔*	*↔*	*↔*	*↔*	*↔*
	Phase 3	*↔*	*↔*	*↔*	↓	*↔*
	Phase 1	—	↓	↓	*↔*	↓
IL-10	Phase 2	*↔*	*↔*	*↔*	*↔*	*↔*
	Phase 3	↓	↓	↓	↓	↓
	Phase 1	—	*↔*	*↔*	*↔*	↑
MCP-1	Phase 2	*↔*	*↔*	*↔*	*↔*	*↔*
	Phase 3	*↔*	*↔*	*↔*	*↔*	*↔*
	Phase 1	—	↓	*↔*	*↔*	*↔*
VCAM-1	Phase 2	*↔*	*↔*	*↔*	*↔*	*↔*
	Phase 3	*↔*	*↔*	*↔*	*↔*	*↔*
	Phase 1	—	*↔*	*↔*	*↔*	*↔*
Cldn3	Phase 2	*↔*	*↔*	*↔*	*↔*	*↔*
	Phase 3	*↔*	↑	*↔*	*↔*	*↔*

Different arrows summarize the effects on gene expression: *↔*: no effect, ↑: increase, ↓: decrease, and n.d.: not detectable. Means ± SEM and the results of statistical analysis are presented in the appendix.

**Table 7:** Effects of feeding broccoli extract and various essential oils on relative mRNA expression of various pro- and anti-inflammatory parameters in colon of DSS-treated rats compared to an untreated control.

Colon	Period	Experimental group compared				
		with untreated control rats				
Gene		DSS	BE	Cuo	To	Ro
	Phase 1	—	*↔*	*↔*	*↔*	*↔*
Nrf2	Phase 2	↓	↓	*↔*	↓	↓
	Phase 3	*↔*	*↔*	*↔*	*↔*	*↔*
	Phase 1	—	*↔*	*↔*	*↔*	↑
Keap1	Phase 2	*↔*	*↔*	*↔*	*↔*	*↔*
	Phase 3	*↔*	*↔*	*↔*	*↔*	*↔*
	Phase 1	—	*↔*	*↔*	*↔*	*↔*
HO1	Phase 2	*↔*	*↔*	*↔*	*↔*	*↔*
	Phase 3	*↔*	*↔*	*↔*	↑	*↔*
	Phase 1	—	*↔*	*↔*	*↔*	*↔*
NQO1	Phase 2	*↔*	*↔*	*↔*	↓	*↔*
	Phase 3	*↔*	*↔*	*↔*	*↔*	*↔*
	Phase 1	—	↓	*↔*	*↔*	*↔*
SOD1	Phase 2	*↔*	*↔*	*↔*	*↔*	*↔*
	Phase 3	*↔*	*↔*	*↔*	*↔*	*↔*
	Phase 1	—	*↔*	*↔*	*↔*	↑
GPx2	Phase 2	*↔*	*↔*	↑	*↔*	*↔*
	Phase 3	↓	↓	↓	↓	↓
	Phase 1	—	↓	*↔*	*↔*	↑
GSTK1	Phase 2	*↔*	*↔*	*↔*	↓	↓
	Phase 3	*↔*	↓	↓	↓	↓
	Phase 1	—	*↔*	*↔*	*↔*	↑
GSTP1	Phase 2	*↔*	*↔*	*↔*	↓	*↔*
	Phase 3	↓	↓	↓	↓	↓
	Phase 1	—	*↔*	↑	*↔*	↑
GSTT2	Phase 2	↓	↓	↓	↓	↓
	Phase 3	*↔*	*↔*	*↔*	*↔*	*↔*

Different arrows summarize the expression results: *↔*: no effect, ↑: increase, and ↓: decrease. For statistically analyzed means ± SEM see Table 10.

**Table 8:** Effects of feeding broccoli extract and various essential oils on relative mRNA expression of various pro- and anti-inflammatory parameters in colon of DSS-treated rats compared to an untreated control. Values are means ± SEM and represent relative mRNA concentrations as *n*-fold of group Con = 1. Different small letters in a row indicate significant differences between means (*P* ≤ 0.05). *n* = 4 in phase 1, *n* = 6 in phase 2 and phase 3.

Colon		Experimental group											
	Period	Con		DSS		BE		Cuo		To		Ro	
Gene		MW	SEM	MW	SEM	MW	SEM	MW	SEM	MW	SEM	MW	SEM
	Phase 1	1.00	0.53	—	—	0.56	0.10	1.04	0.10	0.56	0.21	1.17	0.70
NF*κ*B	Phase 2	1.00	0.20	1.03	0.30	0.36	0.02	1.00	0.44	2.21	1.37	0.51	0.10
	Phase 3	1.00^a^	0.07	3.05^b^	1.23	1.52^ab^	0.28	1.31^ab^	0.30	1.09^ab^	0.16	1.51^ab^	0.42
	Phase 1	1.00	0.45	—	—	0.31	0.05	0.38	0.12	0.42	0.15	0.41	0.14
TNF*α*	Phase 2	1.00^a^	0.15	3.96^ab^	1.48	3.84^ab^	1.28	1.65^ab^	0.59	4.72^b^	1.49	2.98^ab^	0.76
	Phase 3	1.00^a^	0.18	3.71^b^	0.85	4.56^b^	0.97	3.75^b^	0.78	4.18^b^	1.08	3.34^b^	0.90
	Phase 1	1.00	0.81	—	—	0.13	0.03	0.90	0.40	0.55	0.29	0.96	0.61
COX2	Phase 2	1.00^ac^	0.35	17.7^b^	10.26	2.43^ac^	1.00	1.68^ac^	0.71	3.59^bc^	1.10	1.25^ac^	0.42
	Phase 3	1.00	0.17	1.31	0.55	1.41	0.34	0.87	0.27	1.10	0.33	1.00	0.21
	Phase 1	1.00	0.10	—	—	1.48	0.52	1.72	0.45	1.01	0.41	1.60	0.52
IL-1*β*	Phase 2	1.00	0.33	1.10	0.34	1.70	0.82	1.21	0.36	0.52	0.22	1.17	0.29
	Phase 3	1.00^a^	0.33	4.69^b^	1.40	2.32^ab^	0.53	0.96^a^	0.22	2.26^a^	1.01	1.77^a^	1.02
	Phase 1	1.00	0.43	—	—	2.14	1.46	1.95	0.58	2.31	1.28	2.23	1.04
IL-10	Phase 2	1.00^a^	0.39	1.23^ab^	0.35	1.46^ab^	0.31	2.79^b^	0.65	22.5^b^	15.03	3.79^c^	1.50
	Phase 3	n.d.		n.d.		n.d.		n.d.		n.d.		n.d.	
	Phase 1	1.00	0.12	—	—	0.88	0.09	0.94	0.04	1.46	0.45	1.04	0.23
MCP-1	Phase 2	1.00^a^	0.22	1.51^ab^	0.20	2.20^b^	0.30	1.67^b^	0.15	2.76^b^	0.56	2.03^b^	0.58
	Phase 3	1.00^a^	0.27	2.60^bc^	0.47	3.54^b^	0.98	1.10^a^	0.23	0.86^a^	0.26	1.67^ac^	0.57
	Phase 1	1.00^a^	0.42	—	—	0.43^ab^	0.06	0.34^b^	0.10	0.41^b^	0.14	0.38^b^	0.21
VCAM-1	Phase 2	1.00^ab^	0.46	2.40^a^	1.18	0.39^b^	0.19	0.39^b^	0.13	0.62^b^	0.25	0.66^ab^	0.42
	Phase 3	1.00^a^	0.35	6.21^b^	2.20	4.56^b^	0.53	1.97^a^	1.14	1.61^a^	0.79	1.07^a^	0.53
	Phase 1	1.00^a^	0.31	—	—	1.03^a^	0.19	1.71^ab^	0.20	1.63^ab^	0.14	2.37^b^	0.75
Cldn3	Phase 2	1.00^a^	0.38	0.57^ab^	0.21	0.22^b^	0.08	0.49^ab^	0.22	0.25^b^	0.21	0.25^b^	0.07
	Phase 3	1.00^a^	0.18	1.46^ab^	0.34	0.92^a^	0.06	1.07^a^	0.12	1.98^b^	0.36	1.24^a^	0.30

**Table 9:** Effects of feeding broccoli extract and various essential oils on relative mRNA expression of various pro- and anti-inflammatory parameters in the liver of DSS-treated rats compared to an untreated control. Values are means ± SEM and represent relative mRNA concentrations as *n*-fold of group Con = 1. Different small letters in a row indicate significant differences between means (*P* ≤ 0.05). *n* = 4 in phase 1, *n* = 6 in phase 2 and phase 3.

Liver		Experimental group											
Gene	Period	Con		DSS		BE		Cuo		To		Ro	
		MW	SEM	MW	SEM	MW	SEM	MW	SEM	MW	SEM	MW	SEM
	Phase 1	1.00	0.15	—	—	1.06	0.11	0.99	0.06	1.03	0.06	0.97	0.08
NF*κ*B	Phase 2	1.00	0.06	0.94	0.07	0.99	0.03	0.99	0.05	1.02	0.07	1.02	0.06
	Phase 3	1.00	0.04	0.96	0.03	1.00	0.03	0.99	0.04	1.07	0.05	1.06	0.06
	Phase 1	1.00	0.17	—	—	1.04	0.07	1.06	0.12	0.90	0.03	1.10	0.06
TNF*α*	Phase 2	1.00	0.13	1.01	0.14	0.89	0.15	1.04	0.15	0.92	0.14	0.82	0.08
	Phase 3	1.00^a^	0.04	1.71^b^	0.37	1.06^ab^	0.12	1.14^ab^	0.17	1.16^ab^	0.11	1.55^ab^	0.25
	Phase 1	1.00^abc^	0.15	—	—	0.81^ab^	0.09	1.02^ac^	0.06	0.75^b^	0.15	1.12^c^	0.06
COX2	Phase 2	1.00	0.11	1.16	0.20	1.19	0.12	1.34	0.27	1.50	0.25	1.13	0.12
	Phase 3	n.d.		n.d.		n.d.		n.d.		n.d.		n.d.	
	Phase 1	1.00	0.20	—	—	1.00	0.15	1.02	0.16	0.87	0.10	1.13	0.12
IL-1*β*	Phase 2	1.00	0.11	1.04	0.11	0.93	0.12	1.01	0.14	1.12	0.10	0.98	0.08
	Phase 3	1.00^a^	0.03	0.95^a^	0.08	0.85^ab^	0.07	0.84^ab^	0.06	0.75^b^	0.05	1.01^ab^	0.06
	Phase 1	1.00^a^	0.19	—	—	0.67^b^	0.11	0.56^b^	0.08	0.70^ab^	0.15	0.68^b^	0.08
IL-10	Phase 2	1.00	0.12	0.77	0.10	1.15	0.25	1.06	0.15	1.06	0.09	1.01	0.09
	Phase 3	1.00^a^	0.06	0.76^b^	0.05	0.79^b^	0.08	0.67^b^	0.06	0.69^b^	0.03	0.77^b^	0.03
	Phase 1	1.00^a^	0.05	—	—	1.25^ab^	0.17	1.13^a^	0.11	1.18^a^	0.25	1.78^b^	0.23
MCP-1	Phase 2	1.00	0.12	1.00	0.07	1.08	0.15	1.20	0.11	1.12	0.17	1.19	0.13
	Phase 3	1.00	0.07	1.01	0.12	1.00	0.10	0.96	0.11	1.00	0.10	1.01	0.03
	Phase 1	1.00^a^	0.20	—	—	0.74^b^	0.08	0.80^ab^	0.02	0.85^ab^	0.07	0.91^ab^	0.06
VCAM-1	Phase 2	1.00	0.07	1.06	0.12	0.85	0.10	0.98	0.02	1.09	0.07	0.89	0.05
	Phase 3	1.00	0.05	0.89	0.08	1.02	0.11	1.07	0.11	0.92	0.08	1.03	0.09
	Phase 1	1.00	0.19	—	—	1.02	0.30	0.85	0.15	0.91	0.22	0.65	0.07
CRP	Phase 2	1.00	0.20	1.07	0.21	0.74	0.05	0.93	0.20	0.85	0.10	1.14	0.15
	Phase 3	1.00^a^	0.11	0.87^a^	0.11	1.37^b^	0.21	0.89^a^	0.10	0.87^a^	0.12	0.73^a^	0.06

**Table 10:** Effects of feeding broccoli extract and various essential oils on relative mRNA expression of Nrf2, Keap1, and various ARE-regulated enzymes in colon of DSS-treated rats compared to an untreated control. Values are means ± SEM and represent relative mRNA concentrations as *n*-fold of group Con = 1. Different small letters in a row indicate significant differences between means (*P* ≤ 0.05). *n* = 4 in phase 1, *n* = 6 in phase 2 and phase 3.

Colon		Experimental group											
Gene	Period	Con		DSS		BE		Cuo		To		Ro	
		MW	SEM	MW	SEM	MW	SEM	MW	SEM	MW	SEM	MW	SEM
	Phase 1	1.00	0.21	—	—	1.00	0.07	1.31	0.16	1.26	0.19	1.23	0.11
Nrf2	Phase 2	1.00^a^	0.13	0.53^bc^	0.09	0.51^bc^	0.07	0.63^ab^	0.08	0.36^c^	0.07	0.58^bc^	0.11
	Phase 3	1.00	0.28	0.86	0.16	0.89	0.10	1.13	0.13	1.10	0.13	0.69	0.08
	Phase 1	1.00^ab^	0.44	—	—	0.51^a^	0.13	1.04^ab^	0.12	0.87^ab^	0.21	2.44^b^	1.29
Keap1	Phase 2	1.00	0.16	0.93	0.15	1.02	0.18	0.81	0.11	0.67	0.10	0.86	0.09
	Phase 3	1.00	0.19	1.06	0.23	0.69	0.05	1.02	0.29	0.98	0.13	1.43	0.36
	Phase 1	1.00	0.47	—	—	0.48	0.09	1.09	0.35	0.85	0.18	1.40	0.48
HO1	Phase 2	1.00^ab^	0.23	1.71^a^	0.33	1.46^ab^	0.20	1.33^ab^	0.36	0.96^ab^	0.28	0.94^b^	0.29
	Phase 3	1.00^a^	0.17	1.28^a^	0.13	0.99^a^	0.09	1.01^a^	0.18	1.97^b^	0.29	1.04^a^	0.19
	Phase 1	1.00	0.23	—	—	0.54	0.07	0.56	0.13	0.91	0.14	1.05	0.46
NQO1	Phase 2	1.00^ab^	0.26	1.57^a^	0.52	0.94^ab^	0.13	0.85^ab^	0.12	0.59^b^	0.15	0.84^ab^	0.15
	Phase 3	1.00	0.21	1.31	0.36	1.17	0.14	1.10	0.37	2.17	0.44	2.41	1.05
	Phase 1	1.00^a^	0.03	—	—	0.74^b^	0.09	0.94^ab^	0.09	0.95^ab^	0.08	0.78^ab^	0.12
SOD1	Phase 2	1.00	0.12	1.08	0.19	0.80	0.06	1.05	0.12	0.76	0.17	0.92	0.17
	Phase 3	1.00	0.19	1.07	0.09	0.98	0.07	1.04	0.11	1.04	0.07	0.90	0.13
	Phase 1	1.00^ab^	0.29	—	—	0.56^a^	0.05	0.95^ab^	0.23	0.61^a^	0.11	1.43^b^	0.34
GPx2	Phase 2	1.00^a^	0.25	1.49^ab^	0.40	1.16^ab^	0.30	2.36^b^	0.51	1.73^ab^	0.45	1.94^ab^	0.86
	Phase 3	1.00^a^	0.11	0.33^b^	0.06	0.40^b^	0.05	0.46^b^	0.12	0.40^b^	0.11	0.27^b^	0.04
	Phase 1	1.00^a^	0.18	—	—	0.52^b^	0.11	1.19^ac^	0.12	0.90^a^	0.13	1.88^c^	0.45
GSTK1	Phase 2	1.00^a^	0.08	0.70^ab^	0.15	0.75^ab^	0.11	0.90^ab^	0.16	0.53^b^	0.11	0.55^b^	0.11
	Phase 3	1.00^a^	0.05	0.79^ab^	0.18	0.60^b^	0.08	0.70^b^	0.12	0.65^b^	0.09	0.51^b^	0.05
	Phase 1	1.00^ab^	0.14	—	—	0.65^a^	0.13	1.16^bc^	0.20	0.84^ab^	0.14	1.64^c^	0.21
GSTP1	Phase 2	1.00^a^	0.08	0.91^a^	0.21	0.70^a^	0.12	0.72^a^	0.09	0.44^b^	0.16	0.66^a^	0.06
	Phase 3	1.00^a^	0.07	0.47^b^	0.05	0.68^b^	0.09	0.68^b^	0.09	0.63^b^	0.11	0.51^b^	0.08
	Phase 1	1.00^ab^	0.08	—	—	0.65^a^	0.24	1.60^b^	0.21	0.96^a^	0.21	1.58^b^	0.18
GSTT2	Phase 2	1.00^a^	0.12	0.60^b^	0.10	0.43^b^	0.07	0.55^b^	0.09	0.41^b^	0.11	0.49^b^	0.10
	Phase 3	1.00	0.08	2.14	0.40	1.04	0.08	1.13	0.18	1.75	0.24	2.69	0.96

## Appendix

The complete results (means ± SEM) of the mRNA expression analyses, including statistical evaluation, are shown in Tables 8, 9, and 10.
